# A Multiphysics Peridynamic Model for Simulation of Fracture in Si Thin Films during Lithiation/Delithiation Cycles

**DOI:** 10.3390/ma14206081

**Published:** 2021-10-14

**Authors:** Xiaofei Wang, Qi Tong

**Affiliations:** Department of Aeronautics and Astronautics, Fudan University, Shanghai 200433, China; 19110290008@fudan.edu.cn

**Keywords:** lithium-ion batteries, electrodes, silicon thin films, fracture, peridynamics

## Abstract

Material failure is the main obstacle in fulfilling the potential of electrodes in lithium batteries. To date, different failure phenomena observed experimentally in various structures have become challenging to model in numerical simulations. Moreover, their mechanisms are not well understood. To fill the gap, here we develop a coupled chemo-mechanical model based on peridynamics, a particle method that is suitable for simulating spontaneous crack growth, to solve the fracture problems in silicon thin films due to lithiation/delithiation. The model solves mechanical and lithium diffusion problems, respectively, and uses a coupling technique to deal with the interaction between them. The numerical examples of different types of Si films show the advantage of the model in this category and well reproduce the fracture patterns observed in the experiments, demonstrating that it is a promising tool in simulating material failure in electrodes.

## 1. Introduction

Silicon, owing to its high energy capacity, is widely used in lithium-ion batteries as one of the most promising electrode materials. The theoretical capacity can be as high as 4200 mAh/g [1,2]. However, the current technology is still far from reaching this potential in practice. The main challenge resides in the question of how to preserve the capacity after a large number of charging/discharging cycles. The insertion and extraction of lithium can change the volume of silicon by 300% [3], leading to fragmentation of the electrodes and substantial capacity loss [4,5]. Although various nano-structured electrodes, such as nanoparticles [6,7], nanowires [2], and core-shell nanocomposites [8,9,10], have been fabricated to accommodate large deformation and to provide fracture resistance, mechanical degradation is still the main barrier to fulfilling the potential of silicon electrodes. Understanding the fundamental mechanism of the interplay between the lithium diffusion and the mechanical behaviors of these electrodes is desirable in guiding the design of next-generation electrodes.

The past decades have witnessed enormous efforts in studying lithium-induced material failure in silicon electrodes, experimentally and theoretically. Early works, dating back to the late 1990s, identified large volume changes as a major issue in preventing the development of high-capacity electrodes [11,12]. In situ experimental observations have found some evidence of stress evolution and fracture pattern formation due to the lithiation/delithiation process in nanostructured electrodes [13,14,15,16,17]. Theoretical attempts to link the mechanical stress and lithium diffusion were started by Christensen and Newman [18]. Subsequently, extensive models were built to deal with different aspects of the problem, such as the treatment of elastic-plastic material behavior [19,20,21] and the introduction of dislocation [22]. In addition, analytical solutions for various composite nanostructures have been developed, such as core-shell structures [23,24,25,26,27,28]. Despite this progress, in situ experiments are largely restricted in monitoring the dynamic process of chemical reactions and mechanical responses, yet they are expensive and time-consuming. On the other hand, theoretical modeling has met with difficulty in describing material failure, such as the pulverization of the electrodes.

The advancement of numerical methods provides powerful, portable and inexpensive tools for describing and predicting the chemo-mechanical interplay in silicon electrodes. Among them, cohesive zone models integrated into the finite element framework are widely used to consider the coupled problems of lithium diffusion and mechanical deformation [29,30]. Phase field models have also found success in simulating the damage evolution as lithium ions are inserted [31,32]. However, capturing the spontaneous crack growth and revealing the fracture patterns during lithiation/delithiatin cycles are still challenging for the current models. For instance, the simulation of dry bed-lake fractures in silicon thin films [33,34] requires sophisticated tools. The recently developed peridynamics method [35,36,37] provides a promising approach in dealing with complex fracture problems due to chemo-mechanical coupling. The model requires no supplementary criterion for crack growth, which is more convenient in this category compared with other numerical methods. The model has been successfully used in thermal and diffusion-induced deformation and fracture [38,39,40], multiscale crack growth [41,42], etc.

In this work, we aim at establishing a concurrently coupled chemo-mechanical model based on peridynamics to solve fracture problems in lithiated silicon thin films. Section 2 provides the basic theory of the model, including the theory of mechanical behaviors and lithium diffusion, and describes the technique that couples the two fields. Section 3 presents the numerical examples of fractures in silicon thin films due to lithiation/delithiation. Section 4 summarizes the work.

## 2. Method and Model

The peridynamic (PD) theory, introduced as a reformulation of classical continuum mechanics, is developed using integral equations, allowing the expression of damage initiation and propagation at multiple sites with arbitrary paths inside the materials without resorting to special crack growth criteria. In the PD theory, the space area is discretized into a series of material points, with each material point *x* within a certain distance δ. For material points outside this domain, it is assumed that the interactions are weak so that they can be ignored. Hence, all material points inside the domain build up the horizon $\left(H_{x}=\left\{x^{\prime }\in R:\left|x^{\prime }-x\right|\leq \delta \right\}\right)$ of the material point *x*. The lines between material points *x* and $x^{\prime }$ represent PD bonds, transferring the pair-wise information between points. In peridynamic mechanical models, bonds exert forces between points in the horizon, and are used in the formulation for material deformation and damage. In the peridynamic diffusion model, bonds carry concentration information from one point to another.

In this section, we briefly describe the peridynamic mechanical and diffusion models. We then introduce the newly developed chemo-mechanical model to study spontaneous crack growth in silicon thin films during lithiation/delithiation processes.

### 2.1. Bond-Based Peridynamic Mechanical Model

The PD equation of motion of each material point can be written according to Newton’s Second Law as

(1) $$\rho \frac{\partial^{2}u\left(x,t\right)}{\partial t^{2}}=\int_{H_{x}}f\left(u\left(x^{\prime },t\right),u\left(x,t\right),x^{\prime },x,t\right)dV_{x^{\prime }}+b\left(x,t\right)$$

where ρ is the mass density, *u* is the displacement, $dV_{x^{\prime }}$ is the integration variable that indicates the infinitesimal domain located at point $x^{\prime }$, and $H_{x}$ is the horizon, representing the family of particles within a local region centered by *x*. *b* is a prescribed body-force-density field, *f* is the response function which is defined as the force vector per unit volume squared that the material point $x^{\prime }$ exerts on the material point at *x* if their distance is less than the radius δ of the horizon. The undeformed bond vector is defined as $\varepsilon =x^{\prime }-x$, and the relative displacement is $\eta =u^{\prime }-u$. Thus, the deformed bond vector can be calculated as $\eta +\varepsilon =\left(x^{\prime }+u^{\prime }\right)-\left(x+u\right)$.

In the PD theory, the interaction between two material points is described by the force density function. All material-specific information is contained in the force density function that illustrates the dependence between the interparticle forces on the reference positions and the displacements of the points. The displacement of a material point is then integrated based on Equation (1). The force density function for a linear micro-elastic material is given as

(2) $$f\left(\eta ,\varepsilon \right)=\frac{\eta +\varepsilon }{\left|\eta +\varepsilon \right|}u\left(\varepsilon ,t\right)cs$$

where *c* is the micro-modulus function of the bond. For three-dimensional problems, the micro-modulus function of the bond can be defined as

(3) $$c=\frac{12E}{\pi \delta^{4}}$$

Here *E* is Young’s modulus, δ is the size of the horizon. *s* is the stretch that is defined as

(4) $$s=\frac{\left|\eta +\varepsilon \right|-\left|\varepsilon \right|}{\left|\varepsilon \right|}$$

and it is the ratio of the change in distance to the initial distance between points $x^{\prime }$ and *x*. The failure condition of each bond can be represented by a failure parameter as

(5) $$\mu \left(\varepsilon ,t\right)=\left\{\begin{array}{cc} 1 & s<s_{0}\\ 0 & s\geq s_{0} \end{array}\right.$$

where $s_{0}$ is the critical stretch, which can be calculated in terms of the critical energy release rate of a particular material. For three-dimensional problems, the critical stretch of the bond can be defined as

(6) $$s_{0}=\sqrt{\frac{5\Gamma }{9K\delta }}$$

where Γ is the fracture energy and *K* is the bulk modulus. When the stretch of the bond between two points exceeds the critical stretch $s_{0}$, the bond breaks, and these two points cease to interact with each other. Once the bond breaks, the load on material point *x* will be redistributed to the remaining bonds and the deformation state changes. Therefore, damage is treated as part of the constitutive model through the irreversible breakage of interactions. In order to quantify the damage state of a material point, a local damage parameter of a material point is defined as

(7) $$\varphi \left(x,t\right)=1-\frac{\int_{H(x)}\mu \left(\varepsilon ,t\right)dV}{\int_{H(x)}dV}$$

Local damage is the percentage of broken bonds associated with the material point *x* and varies from 0 to 1, where 0 represents undamaged and 1 refers to fully damaged cases.

### 2.2. Peridynamic Diffusion Model

In order to model lithium diffusion, we introduce the diffusion equation that characterize the mass transport

(8) $$\frac{\partial C}{\partial t}+\nabla \cdot J=0$$

where the Li ion concentration *C* is the number of Li atoms per unit volume. The boundary value is represented by $C_{0}$. The flux of the lithium ions *J* is the number of Li atoms per unit time crossing a unit area, which consists of two contributions in a chemo-mechanical coupled environment [43]. The first term is due to the concentration gradient,

(9) $$J_{Li,c}=-D_{0}\nabla C$$

The other contribution is from the stress,

(10) $$J_{Li,\sigma }=D_{0}\left(1-\frac{C}{C_{max}}\right)\frac{\Omega_{Li}C}{RT}\nabla \sigma$$

The hydrostatic stress gradient that drives Li mass transport reaches zero at both $C_{0}=0$ and $C_{0}=C_{max}$. In the above equations, $D_{0}$ is the diffusivity of lithium ions, Ω is the partial molar volume of Li in silicon, *k* is the gas constant, *T* is the temperature, and $\sigma_{m}=\left(\sigma_{1}+\sigma_{2}+\sigma_{3}\right)/3$ is the bulk stress, with $\sigma_{1}$, $\sigma_{2}$, and $\sigma_{3}$ being diagonal terms of the Cauchy stress tensor. The Cauchy stress tensor is calculated as

(11) $$\sigma =\frac{1}{2}\int_{H(x)}f\left(\eta ,\varepsilon \right)⊗\left(\eta +\varepsilon \right)dV_{x^{\prime }}$$

The dynamic process of lithium diffusion is represented by Fick’s second law. Note that the stress induced by a large volume change during cycling cannot be ignored. By considering the concentration gradient and hydrostatic stress gradient, Fick’s second law is modified as

(12) $$\frac{\partial C(x,t)}{\partial t}=D_{0}\frac{\partial^{2}C}{\partial r^{2}}-\frac{D_{0}\Omega }{kT}\left[C\left(1-\frac{C}{C_{max}}\right)\frac{\partial^{2}\sigma_{m}}{\partial r^{2}}+\left(1-\frac{2C}{C_{max}}\right)\frac{\partial \sigma_{m}}{\partial r}\times \frac{\partial C}{\partial r}\right]$$

Although peridynamics was originally introduced for deformation fields, it is also applicable to other fields, including diffusion. However, it is not straightforward to express the coupled diffusion equation in a peridynamic form. In this study, this has been achieved using the concept of the peridynamic differential operator [44,45,46]. All spatial derivatives in the classical diffusion equation can be transformed into peridynamic form as

(13) $$\frac{\partial^{2}C\left(x,t\right)}{\partial r^{2}}=\left(\frac{6}{\pi \delta^{4}}\right)\int_{H(x)}\frac{C\left(x^{\prime },t\right)-C\left(x,t\right)}{\left|\varepsilon \right|}dV_{x^{\prime }}$$

(14) $$\frac{\partial^{2}\sigma_{m}\left(x,t\right)}{\partial r^{2}}=\left(\frac{6}{\pi \delta^{4}}\right)\int_{H(x)}\frac{\sigma_{m}\left(x^{\prime },t\right)-\sigma_{m}\left(x,t\right)}{\left|\varepsilon \right|}dV_{x^{\prime }}$$

(15) $$\begin{array}{cc} \frac{\partial \sigma_{m}\left(x,t\right)}{\partial r}\times \frac{\partial C(x,t)}{\partial r}= & \left(\frac{81}{16\pi^{2}\delta^{6}}\right)\left(\int_{H(x)}\frac{\sigma_{m}\left(x^{\prime },t\right)-\sigma_{m}\left(x,t\right)}{\left|\varepsilon \right|}dV_{x^{\prime }}\right)\\ \; & \times \left(\int_{H(x)}\frac{C\left(x^{\prime },t\right)-C\left(x,t\right)}{\left|\varepsilon \right|}dV_{x^{\prime }}\right) \end{array}$$

Therefore, the general Fick’s second law given in Equation (12) can be written in peridynamic form as

(16) $$\begin{array}{cc} \frac{\partial C(x,t)}{\partial t}= & D_{0}\frac{\partial^{2}C}{\partial r^{2}}-\frac{D_{0}\Omega }{kT}\left[C\left(1-\frac{C}{C_{max}}\right)\frac{\partial^{2}\sigma_{m}}{\partial r^{2}}+\left(1-\frac{2C}{C_{max}}\right)\frac{\partial \sigma_{m}}{\partial r}\times \frac{\partial C}{\partial r}\right]\\ = & \left(\frac{6D_{0}}{\pi \delta^{4}}\right)\int_{H(x)}\frac{C\left(x^{\prime },t\right)-C\left(x,t\right)}{|x^{\prime }-x|}dV_{x^{\prime }}\\ \; & -\left(\frac{6D_{0}\Omega }{\pi \delta^{4}kT}\right)\int_{H(x)}C\left(x,t\right)\left(1-\frac{C(x,t)}{C_{max}}\right)\frac{\sigma_{m}\left(x^{\prime },t\right)-\sigma_{m}\left(x,t\right)}{|x^{\prime }-x|}dV_{x^{\prime }}\\ \; & -\left(\frac{81D_{0}\Omega }{16\pi^{2}\delta^{6}kT}\right)\left(\int_{H(x)}\frac{\sigma_{m}\left(x^{\prime },t\right)-\sigma_{m}\left(x,t\right)}{|x^{\prime }-x|}dV_{x^{\prime }}\right)\\ \; & \times \left(\int_{H(x)}\left(1-\frac{2C(x,t)}{C_{max}}\right)\frac{C\left(x^{\prime },t\right)-C\left(x,t\right)}{|x^{\prime }-x|}dV_{x^{\prime }}\right) \end{array}$$

### 2.3. Coupled Peridynamic Chemo-Mechanical Model

This study also extends the peridynamic theory to include the effect of chemo-mechanical loading. Attributed to the insertion of lithium ions, the silicon electrodes experience huge volume changes during the processes of lithiation and delithiation. Therefore, it is necessary to modify the force density function and the failure parameter, i.e., to subtract the volume expansion caused by the intercalation of lithium ions. Without considering the influence of Li-ion concentration, the stretch of the bond between the two material points is s=(|η+ε|−|ε|)/|ε|. When the influence of the Li ion concentration is considered, lithiation-induced linear strain is

(17) $$s_{Li}=\frac{1}{3}\log\left(1+\frac{\Omega \left(C\left(x,t\right)+C\left(x^{\prime },t\right)\right)}{2}\right)$$

Furthermore, a new response function is proposed to include the effects of chemical loading and the distance between the points as

(18) $$\begin{array}{cc} f(\eta ,\varepsilon ) & =\frac{\eta +\varepsilon }{\left|\eta +\varepsilon \right|}\mu \left(\varepsilon ,t\right)c\left(s-s_{Li}\right)\\ \; & =\frac{\eta +\varepsilon }{\left|\eta +\varepsilon \right|}\mu \left(\varepsilon ,t\right)c\left(\frac{\left|\eta +\varepsilon \right|-\left|\varepsilon \right|}{\left|\varepsilon \right|}-\frac{1}{3}\log\left(1+\frac{\Omega \left(C\left(x,t\right)+C\left(x^{\prime },t\right)\right)}{2}\right)\right) \end{array}$$

Similarly, the effect of chemical loading is also included in the failure parameter by extending its definition, given in Equation (5), as

(19) $$\mu \left(\varepsilon ,t\right)=\left\{\begin{array}{cc} 1 & \frac{\left|\eta +\varepsilon \right|-\left|\varepsilon \right|}{\left|\varepsilon \right|}-\frac{1}{3}\log\left(1+\frac{\Omega \left(C\left(x,t\right)+C\left(x^{\prime },t\right)\right)}{2}\right)<s_{0}\\ 0 & \frac{\left|\eta +\varepsilon \right|-\left|\varepsilon \right|}{\left|\varepsilon \right|}-\frac{1}{3}\log\left(1+\frac{\Omega \left(C\left(x,t\right)+C\left(x^{\prime },t\right)\right)}{2}\right)\geq s_{0} \end{array}\right.$$

in which $s_{0}$ is still the critical stretch upon failure. However, the failure between two points occurs when the elastic stretch $s_{0}-s_{Li}$, rather than stretch *s*, exceeds the critical stretch $s_{0}$.

### 2.4. Flowchart of the Numerical Scheme

**Figure 1:**
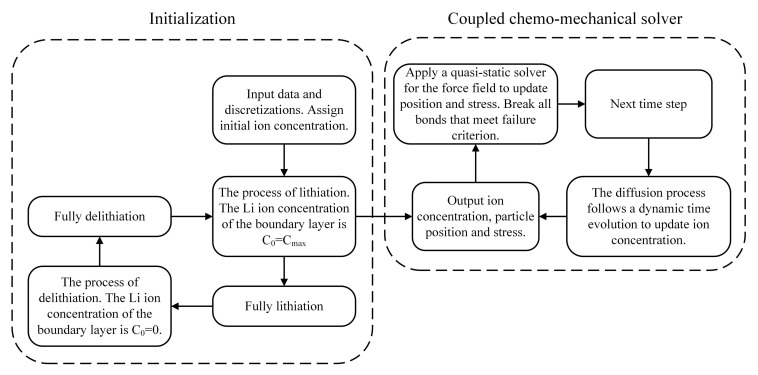
Flowchart of the numerical scheme.

To simulate the spontaneous crack growth in Si thin films, we developed the algorithm shown in Figure 1. The coupled chemo-mechanical model features a large time-scale separation between the diffusion and the fracture processes. Typically, the time scale of the diffusion process is several orders higher than that of the force field. To reduce the computational cost, we employ a quasi-static solver for the force field, whereas the diffusion follows a dynamic time evolution.

## 3. Numerical Examples

### 3.1. Numerical Model

**Figure 2:**
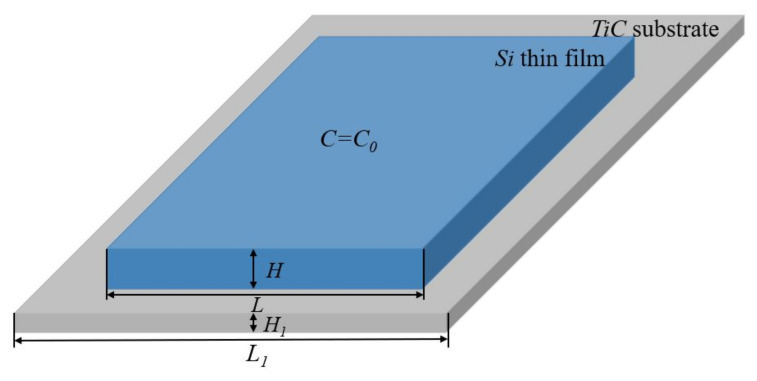
The Si thin film was deposited on the TiC substrate and the boundary conditions for the analysis of the Si thin film in the processes of lithiation and delithiation.

The model investigated is an Si thin film deposited on the TiC substrate, as shown in Figure 2. The Si thin film has the width *L* and the thickness *H*. The TiC substrate has the width $L_{1}$ and the thickness $H_{1}$. The lithium ions are allowed to penetrate the Si thin film over the entire surface that is in contact with the electrolyte. A prescribed constant concentration of lithium ions is considered over the upper free surface. The free surface in contact with the electrolyte has a stress-free condition applied, allowing for volume expansion, whereas the other surface is deposited on the TiC substrate, restricted by the interface. In its present form, the resolution of the diffusion of the lithium ions into the Si thin film is not coupled with its mechanical state, meaning that stress does not assist the ions’ diffusion. The boundary conditions of the Si thin film are illustrated in Figure 2. The diffusion coefficient of lithium ions in the silicon is $2.5\times 10^{-17}$ m${}^{2}$/s. The Young’s moduli of the lithiated silicon and the TiC substrate are $E_{Si}=12$ GPa and $E_{TiC}=439.4$ GPa, respectively. An effective modulus is introduced to account for the interface, given by $1/E_{e}=\left(1/E_{Si}+1/E_{TiC}\right)/2$. The fracture energy is $\Gamma_{f}=2000$ J/m${}^{2}$ for silicon in the first lithiation. During the delithiation process, which consists of broken atomic bonds, the fracture energy was taken to be five times smaller than that during lithiation, i.e., $\Gamma_{f}=400$ J/m${}^{2}$ for silicon in the first delithiation. Furthermore, $\Gamma_{d}=500$ J/m${}^{2}$ is used for the Si−TiC interface. To model the progressive degradation of the material properties after several cycles, the fracture energy $\Gamma_{f}$ is decreased by 9% of its initial value after each cycle of lithiation and delithiation. In the tenth cycle, the fracture energy has been decreased by about 80% of its initial value. The complete list of the parameters of the materials and the numerical model is provided in Table 1.

**Figure 3:**
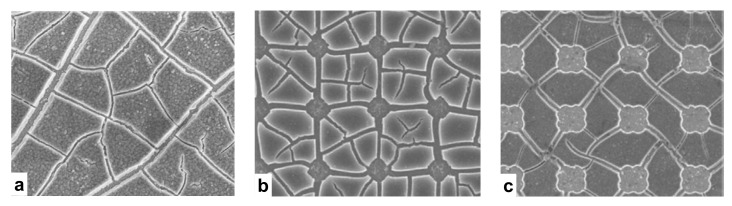
Fracture patterns in (**a**) continuous silicon thin film, (**b**,**c**) patterned films. Reproduced with permission [32].

Experimental observations of the fracture patterns in different silicon membranes are shown in Figure 3. The most common fracture pattern is dry lake-bed cracks [32], which is illustrated in Figure 3a. This type of fracture is typically observed in continuous flat membranes. Since the in-plane stress is isotropic during volume expansion when lithium ions are inserted, cracks grow without a favorable direction. As a result, the crack paths are fairly random. Other types of membranes, shown in Figure 3b,c, are patterned with holes to accommodate volume expansion and to mitigate internal stress. The fracture patterns are more regular and usually crosslink the holes. The proposed multiphysics model was validated in these membranes. The experimental techniques were found in the literature, e.g., [32]. We followed similar procedures in the simulations.

### 3.2. Fracture of Continuous Membrane

**Figure 4:**
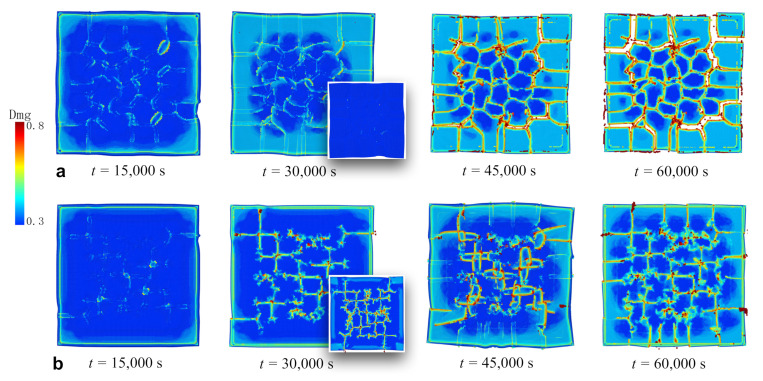
The processes of crack formation and propagation in the continuous Si film during lithiation/delithiation cycles. (**a**) Snapshots in the case of one cycle in 60,000 s. (**b**) Snapshots in the case of two cycles in 60,000 s. The snapshots depict the view from the interface on the bottom of the film. The insets at *t* = 30,000 s are snapshots on the top free surface at the same moments. Colors represent the degrees of damage.

Figure 4 shows snapshots of crack formation and propagation in the continuous silicon membrane during lithiation/delithiation cycles. To investigate the effect of the charging/discharging rate, we considered the cases including one and two cycles in 60,000 s, respectively. Note that the fracture energy $\Gamma_{f}$ decreased by 9% in each cycle because of the material degradation. Figure 4a presents the case with one cycle in 60,000 s. The snapshots are viewed from the interface instead of the free surface to better observe the cracks. The particles are colored based on the damage parameter in Equation (7). The process of lithiation was in the period of 0–30,000 s. Crack initiation is observed at *t* = 15,000 s at some random locations. The volume expansion led to partial detachment of the film from the substrate, through breaking the bonds between the particles in the film and the substrate. As a result, the contour of the film became irregular due to the combined effect of detachment and volume expansion. By the end of the lithiation at *t* = 30,000 s, apparent crack lines were formed, and debonding was propagated inward from each side observed from the damage profile. The inset at *t* = 30,000 s shows the free surface at the same time. It seems that the cracks did not propagate through the thickness direction of the film during lithiation. This was the consequence of the bending upward of the film, where the interface was mostly subjected to tensile stress and the free surface was compressed, where cracks were suppressed. The process of delithiation was in the period of 30,000–60,000 s. The crack segments formed during lithiation further propagated to connect with each other. A crack network had formed at *t* = 45,000 s on the interface and started to travel through the thickness direction of the film. By the end of the cycle at *t* = 60,000 s, apparent fragmentation through the thickness direction of the film could be observed. The overall fracture pattern was isotropic without a preferable crack direction, which corresponds to the dry lake-bed cracks in Figure 3a.

Figure 4b further illustrates the case of two cycles in 60,000 s. Each lithiation or delithiation took 15,000 s. Apparent differences were found as the charging/discharging rate was doubled. By the end of the first cycle at *t* = 30,000 s, crack segments formed but did not crosslink. This feature can be attributed to the faster charging/discharging rate that released and redistributed the internal stress more frequently. Redistribution of stress provided a tendency to form more bifurcations instead of directly linking to each other. In contrast, in the previous case, the lithiation process took 30,000 s, which was enough for the crack segments to become connected under high stress intensity. On the other hand, observable in the insets (free surface) at *t* = 30,000 s, the cracks had already traveled through the thickness direction of the film. This can be compared with the snapshot at the same time in the previous case, where the free surface was still undamaged. Therefore, a complete cycle of lithiation/delithiation was desirable to provide enough tensile stress for the propagating of the cracks through the thickness direction. This was evidenced at *t* = 60,000 s in the previous case. If the cycles kept running, more and more bifurcations would be generated due to the degradation of the materials.

### 3.3. Fracture of Membrane with Square Holes

**Figure 5:**
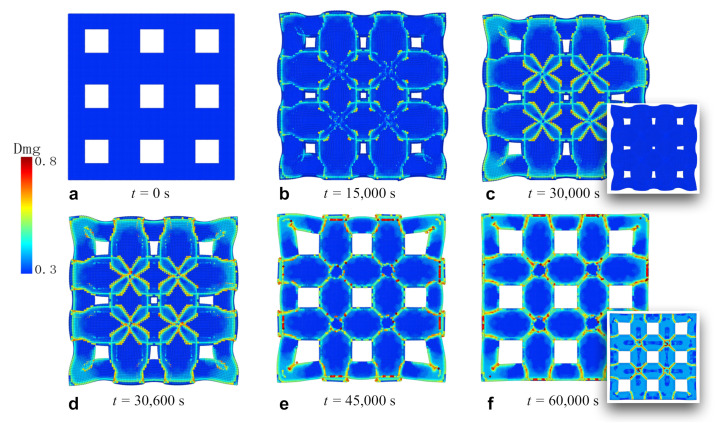
The process of crack formation and propagation in the silicon film pattered with square holes. The holes are located at 25 μm, 75 μm, and 125 μm in each direction from the corners. The width of the holes is 25 μm. The figures and the insets correspond to the same process of lithiation/delithiation as in the continuous film.

To study the fracture in the patterned Si thin films in Figure 3b,c, we created a new sample with nine square holes evenly distributed at 25 μm, 75 μm, and 125 μm in each direction from the corners, as shown in Figure 5a. The width of the squares was 25 μm. The snapshots were taken from the interface, as in the continuous film. The damage profiles on the top free surface are also presented in the insets at the end of the lithiation/delithiation in Figure 5c,f. The coloring method is the same as before.

Several implications can be drawn from the period of lithiation shown in Figure 5b,c. First, the holes were greatly contracted compared with the original configuration. This is the advantage of this structure, where the holes can accommodate the strain caused by lithium-ion insertion through the expansion inward. Therefore, internal stress was mitigated. Second, cracks and debonding formed more easily at the corners of the film as well as the holes due to stress concentration. The initial cracks tended to connect to each other, resulting in diagonally crosslinked crack paths. Third, in contrast to the continuous film, the patterned film demonstrated a regular fracture pattern and a deformed shape. As was the case with the continuous film, cracks were initiated on the bottom interface and did not travel through the film in the thickness direction during lithiation, which is evidenced in the inset in Figure 5c. The followed delithiation process is illustrated in Figure 5d,f. As the lithium ions were extracted from the film, the shape of the film recovered, and the holes turned back to their original sizes. However, the damage was intensified as the cracks walked through the film, as demonstrated in the inset in Figure 5f. The diagonalized fracture pattern can be compared with the experimental results in Figure 3c.

### 3.4. Fracture of Membrane with Circular Holes

**Figure 6:**
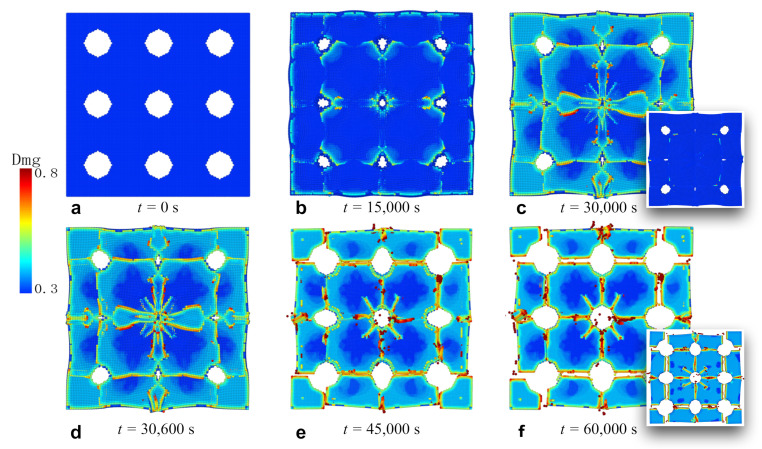
The process of crack formation and propagation in the silicon film pattered with circular holes. The holes were located at 25 μm, 75 μm, and 125 μm in each direction from the corners. The diameter of the holes was 25 μm. The figures and the insets correspond to the same process of lithiation/delithiation as in the continuous film.

The third numerical simulation was conducted based on a silicon thin film patterned with circular holes. The locations and the sizes of the holes were the same as the square holes in the previous sample. The lithiation/delithiation processes are illustrated in Figure 6, where a–c depict the lithiation from 0 to 30,000 s, and d–f present the period of delithiation. Similarly to the continuous and square-patterned films, the cracks initiated and propagated from the interface during lithiation and did not run through the thickness direction. The holes also experienced significant shrinkage as holes of irregular shape and different sizes. However, the cracks grew along the horizontal and vertical directions, and then developed a network by connecting to each other. Since the circular holes do not have sharp corners to induce stress concentration, diagonal connections are not favorable compared with the shorter lines in horizontal and vertical directions. It is noteworthy that the hole in the middle produced cracks along the diagonal directions, but these cracks did not fully expand and connect. Compared with the film with square holes, circular holes provide better accommodation for volume expansion. Throughout the lithiation/delithiation cycle, the overall shape was largely conserved, without significant distortion.

## 4. Conclusions

In summary, we have developed a concurrently coupled chemo-mechanical model based on peridynamics to simulate the complex fracture problems in different types of silicon thin films due to the insertion and extraction of lithium ions. The framework consists of a classic bond-based peridynamic mechanical model, a diffusion model and a coupling technique to reflect the interaction between them. We have used the method to perform numerical simulations of three different thin films, i.e., continuous film and films patterned with square and circular holes, which are typically used to mitigate volume expansion and to reduce the risk of material failure. The continuous film displayed a dry lake-bed type fracture pattern, i.e., an isotropic crack network without a favorable direction. We also investigated the influence of the charging/discharging rate and revealed that a higher rate resulted in more bifurcations because the internal stress was released and redistributed more frequently. The cracks in the patterned films formed networks that connected the holes. The square holes and circular holes experienced different styles of connection, which were diagonal or horizontal/vertical. The simulations reproduced the fracture patterns observed in experiments, which proves the capability of the model to deal with the problems of material failure in electrodes, and to provide a powerful tool to aid in the future design of new structures. Future work includes the applications of the model in different structures and materials, such as spherical particles, hollow core–shell nanotubes, Si electrodes that contain carbon additives and polymer binders, etc. It is also desirable to incorporate complex constitutive models into the framework to study the influence of crystallography.

## Figures and Tables

**Table 1:** Parameters of the materials and the numerical model.

Parameter	Symbol	Value	Units
Diffusion coefficient	$D_{0}$	$2.5\times 10^{-17}$	m${}^{2}$/s
Young’s modulus of lithiated Si	$E_{Si}$	12	GPa
Young’s modulus of TiC	$E_{TiC}$	439.4	GPa
Young’s modulus of Si-TiC	$E_{e}=\frac{2}{\left(\frac{1}{E_{Si}}+\frac{1}{E_{TiC}}\right)}$	23.36	GPa
Poisson’s ratio	υ	0.25	
Fracture energy of Si in first lithiation	$\Gamma_{f}$	2000	J/m${}^{2}$
Fracture energy of Si in first delithiation	$\Gamma_{f}$	400	J/m${}^{2}$
Fracture energy of Si-TiC	$\Gamma_{d}$	500	J/m${}^{2}$
Partial molar volume of Si	Ω	$1.2052\times 10^{-5}$	m${}^{3}$/mol
Gas constant	*k*	8.314	
Temperature	*T*	300	K
Width of Si thin film	*L*	$1.6\times 10^{4}$	nm
Thickness of Si thin film	*H*	600	nm
Width of TiC substrate	$L_{1}$	$2.0\times 10^{4}$	nm
Thickness of TiC substrate	$H_{1}$	300	nm
Grid size	Δ	100	nm
Horizon	δ	300	nm
Critical stretch of Si in first lithiation	$s_{0}=\sqrt{\frac{5\Gamma_{f}}{9K_{Si}\delta }}$	0.68	
Critical stretch of Si in first delithiation	$s_{0}=\sqrt{\frac{5\Gamma_{f}}{9K_{Si}\delta }}$	0.304	
Critical stretch of Si-TiC	$s_{0}=\sqrt{\frac{5\Gamma_{d}}{9K_{e}\delta }}$	0.244	

## Data Availability

The related data are available upon request.
